# Metric learning for image-based flower cultivars identification

**DOI:** 10.1186/s13007-021-00767-w

**Published:** 2021-06-22

**Authors:** Ruisong Zhang, Ye Tian, Junmei Zhang, Silan Dai, Xiaogai Hou, Jue Wang, Qi Guo

**Affiliations:** 1 College of Technology, Beijing Forestry University, Beijing, 100083 China; 2 College of Landscape Architecture, Beijing Forestry University, Beijing, 100083 China; 3 College of Agriculture / College of Tree Peony, Henan University of Science and Technology, Luoyang, 471023 China

**Keywords:** Flower cultivars identification, Metric learning, Center loss, Deep Learning

## Abstract

**Background:**

The study of plant phenotype by deep learning has received increased interest in recent years, which impressive progress has been made in the fields of plant breeding. Deep learning extremely relies on a large amount of training data to extract and recognize target features in the field of plant phenotype classification and recognition tasks. However, for some flower cultivars identification tasks with a huge number of cultivars, it is difficult for traditional deep learning methods to achieve better recognition results with limited sample data. Thus, a method based on metric learning for flower cultivars identification is proposed to solve this problem.

**Results:**

We added center loss to the classification network to make inter-class samples disperse and intra-class samples compact, the script of ResNet18, ResNet50, and DenseNet121 were used for feature extraction. To evaluate the effectiveness of the proposed method, a public dataset Oxford 102 Flowers dataset and two novel datasets constructed by us are chosen. For the method of joint supervision of center loss and *L*_*2*_-softmax loss, the test accuracy rate is 91.88%, 97.34%, and 99.82% across three datasets, respectively. Feature distribution observed by T-distributed stochastic neighbor embedding (T-SNE) verifies the effectiveness of the method presented above.

**Conclusions:**

An efficient metric learning method has been described for flower cultivars identification task, which not only provides high recognition rates but also makes the feature extracted from the recognition network interpretable. This study demonstrated that the proposed method provides new ideas for the application of a small amount of data in the field of identification, and has important reference significance for the flower cultivars identification research.

## Background

As a popular category of plants, cultivars identification of ornamental plants is an important basis for reproduction, cultivation, application, and breeding. With the rapid development of Artificial Intelligence (AI), the study of plant phenotype has made a series of important progress [1–8] as a science of studying plant growth, performance, and composition. Image processing methods based on computer vision are increasingly applied to the study of plant phenotype. Bonnet [3] evaluating how state-of-art computer vision systems do perform in identifying plants compared to human expertise. The results show that the machine can clearly outperform beginners and inexperienced test subjects, which proves the feasibility of identifying plants based on computer vision.

**Fig. 1:**
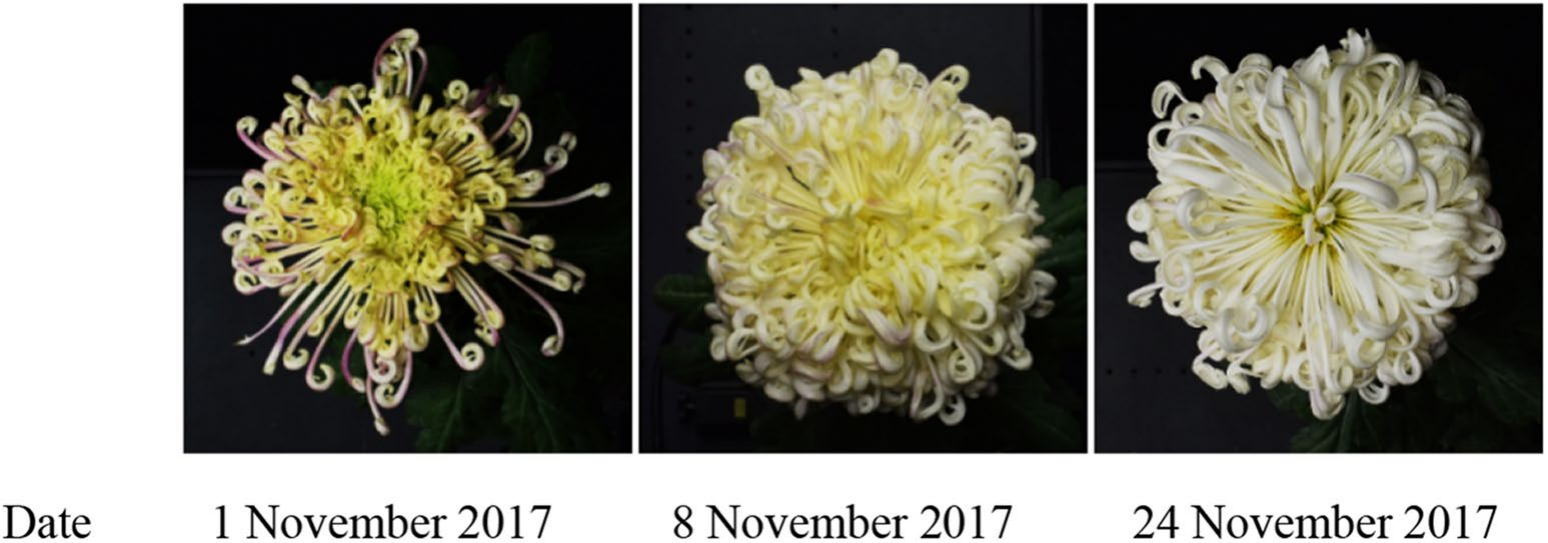
Opening process of one large-flowered chrysanthemum. Figure is from [14], with permission from the rights holder

Flowers with ornamental phenotype have brought a pleasing visual feast to humans due to their unique shapes, rich colors, and varied textures, which is important for flower phenotype research through computer vision. Recent studies have applied deep learning-based methods to flower recognition and made a series of important progress [9–14]. In particular, Lee [9] studied Convolutional Neural Networks (CNN) to learn unsupervised feature representations for 44 different plant species. Deep Convolutional Neural Networks (DCNN) based hybrid method is applied to flower species classification on Flower17 and Flower102 datasets in [13]. Liu [14] proposed a method of large-flowered chrysanthemum cultivar recognition. In the context of deep learning [15], at least thousands of training samples are required for each class to saturate the performance of DCNN on known classes. However, in practice, due to privacy protection restrictions, it is difficult to have a large amount of labeled samples, such as face recognition. For ornamental plants, not all angles have research value, which like the task of face recognition that a complete frontal view of people is required. Due to the limitation of the viewing angle, both tasks are difficult to obtain a large number of variable data for training. Such as chrysanthemum, only one or a few images of each cultivar is effective for further research despite numerous cultivars. Besides, due to the poor generalization ability of the classification neural network, it is difficult for the model to learn to identify new cultivars in the lack of labeled samples. On the other hand, sometimes it is difficult to label samples. Some cultivars’ phenotype changed dramatically during their flower opening process [14], if labeled as the same cultivar will cause ambiguity, as shown in Fig. 1. These problems restrict the application of deep learning in plant phenotype.

Inspired by human rapid learning ability, a challenging machine learning field called Few-Shot Learning (FSL) emerges [16], which helps to relieve the burden of collecting large-scale supervised data. The methods of FSL are being widely applied to various research areas such as computer vision, natural language processing, audio and speech, and data analysis, etc. [17]. Early research of FSL approaches focused on the image field, the solution of image recognition tasks by FSL is that the model can learn quickly for new classes after learning a large amount of data in a certain class. Siamese Nets [18] is the first work that brings deep neural networks into FSL tasks, which consists of twin CNNs that share the same weights. By accepting a pair of samples as inputs, outputs of twin CNNs at the top layer are combined in order to output a single pairwise similarity score. Li [19] proposed an end-to-end deep architecture, Covariance Metric Networks, used in generic few-shot image classification and fine-grained few-shot image classification. The method of metric learning has been widely used in the image field.

Metric learning belongs to FSL. Through a spatial mapping approach that can be learned, an embedding space is obtained, in which all samples are converted into embedding vectors. In embedding space, the distance distribution between samples is being modeled so that similar samples are closer, and vice versa. This approach is applied in many fields, such as image retrieval, face recognition, target tracking, etc. Consequently, metric learning is suitable for the task of flower cultivars identification.

In this work, by using metric learning method, unknown chrysanthemum cultivars were identified. We trained a chrysanthemum cultivars identification system using DCNN which was to transform the image into the embedding space. A DCNN was trained as a classification task where the network learned to classify a given chrysanthemum cultivars image to its correct identity label. But different from the usual classifier training, in addition to the classification loss, it also increases the distance loss. In the embedding space, the classification loss distinguishes different classes, while the distance loss makes similar samples closer. Through the learning of the embedding space, the finally obtained embedding vector has the ability to measure, and the vector can be used for unknown chrysanthemum cultivars identification. To our knowledge, different datasets have a built-in bias, which has a certain impact on the recognition results. Thus, we evaluate the performance of the proposed methods on three datasets, a public dataset and two novel datasets constructed by us. The public dataset Oxford 102 Flowers consists of 102 flower classes, the number of images in each class between 40 and 258. Peony dataset contains 1255 images of 80 cultivars that were manually photographed in 2019. Chrysanthemum dataset contains 2520 images of 126 cultivars that were acquired by an automatic image acquisition device in 2018. The ROC curve, Top-1, and Top-5 evaluation indicators are used to evaluate the model, and the feature distribution of cultivars is visualized by T-SNE.

## Methods

### Chrysanthemum dataset

The image datasets of this research come from traditional Chinese large-flowered chrysanthemum cultivated by the research team of Beijing Forestry University (Beijing, China) in Dadongliu Nursery, Beijing. Cultivar naming standard is referred to Chinese Chrysanthemum Book [20]. The cultivation in our experiment belongs to single-flower cultivation, and the morphologies in these cultivars are similar. The cultivation process follows [14]. All images from chrysanthemum dataset were collected using the chrysanthemum image acquisition device. And the device and image acquisition process are the same as [14]. All the collected pictures were accurately and uniformly marked by the researchers using labelImg v1.7.0 software.

**Fig. 2:**
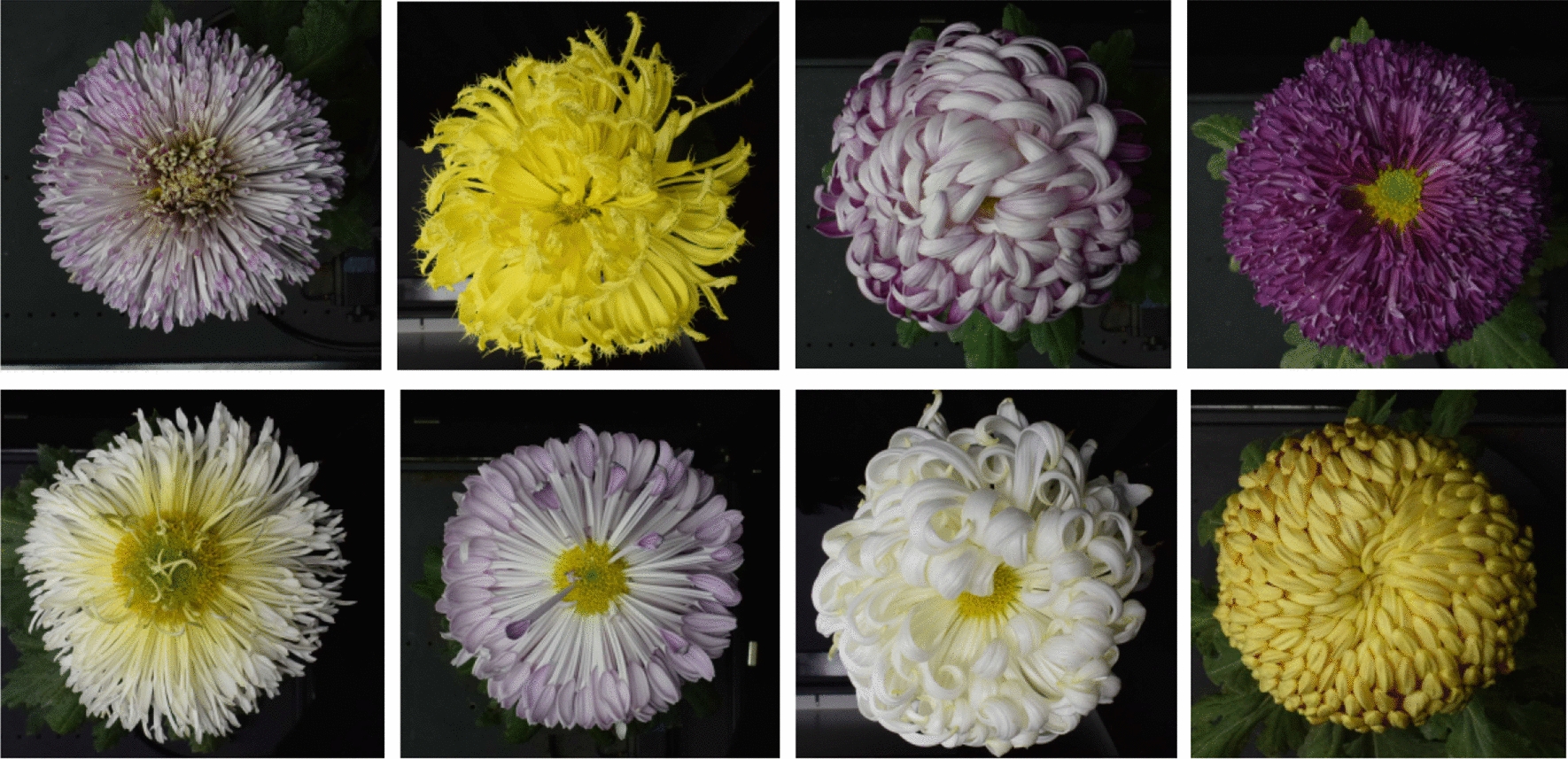
Sample images in chrysanthemum dataset

Images of 126 cultivars were gathered by automatic image acquisition device in 2018, 100 cultivars were randomly selected for training and 26 cultivars for test. In order to balance the samples, we randomly selected 20 images for each cultivar. The angles of the obtained images were consistent with the background, and some of the images were shown in Fig. 2.

**Fig. 3:**
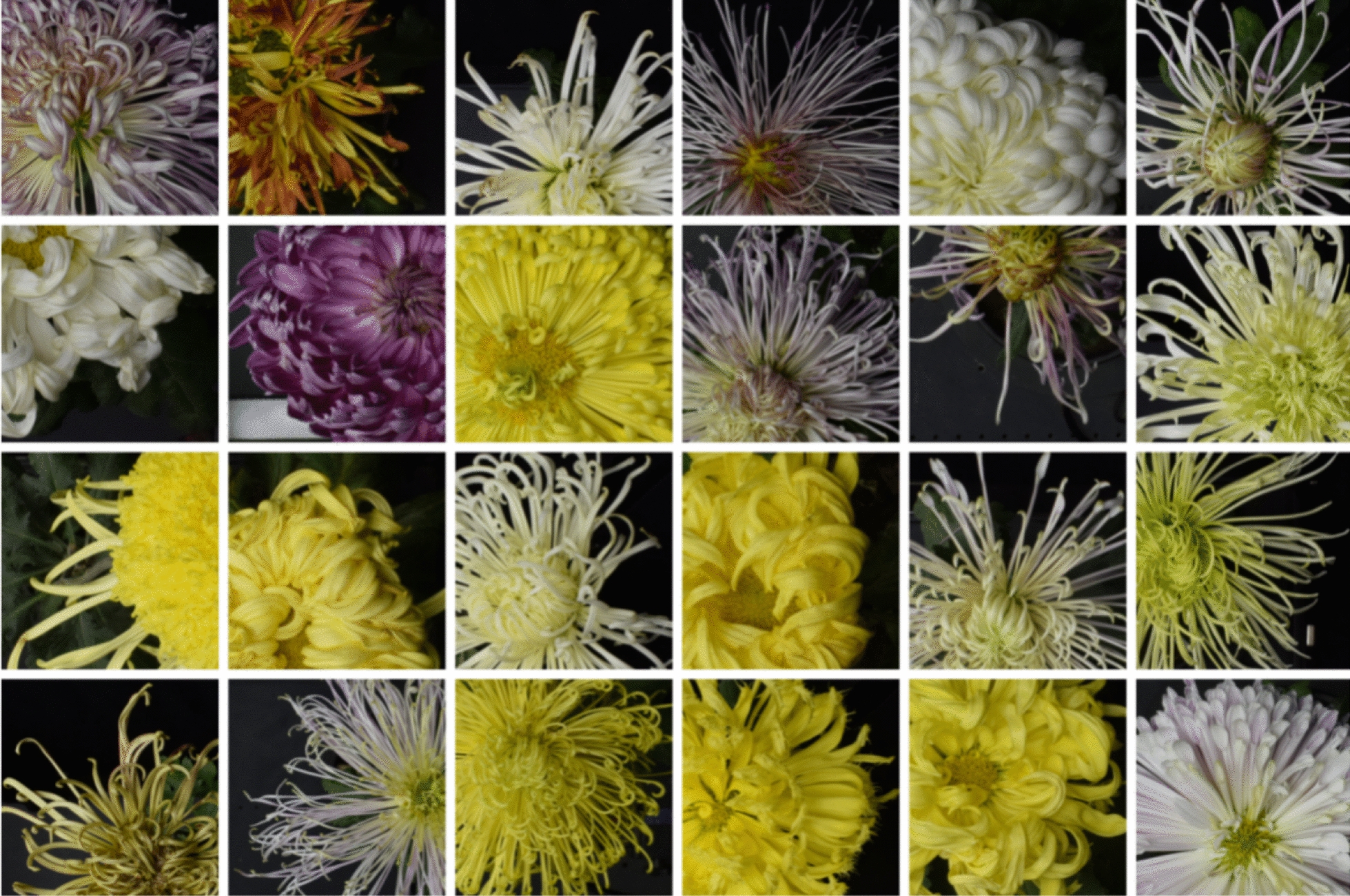
Sample images in chrysanthemum dataset after random cropping

According to [14], DCNN model paid substantial attention to inflorescence edge areas and disc floret areas, which were the key recognition positions. Therefore, DCNN models usually focus on the local information of the image when using deep learning to classify chrysanthemum images. SimCLR [23] has shown that focusing on the part of the image can obtain better recognition results than focusing on the overall image because the overall information of the image is more demanding than the local information. Therefore, to make the model focuses on local information by learning the embedding vector representation of a patch of an image, all the patches of the same image have similar representations, and different images have different representations. By random cropping, we expanded the number of original images by 10 times to a total of 25200 to construct the chrysanthemum dataset. Some of the images were shown in Fig. 3.

### Peony dataset

**Fig. 4:**
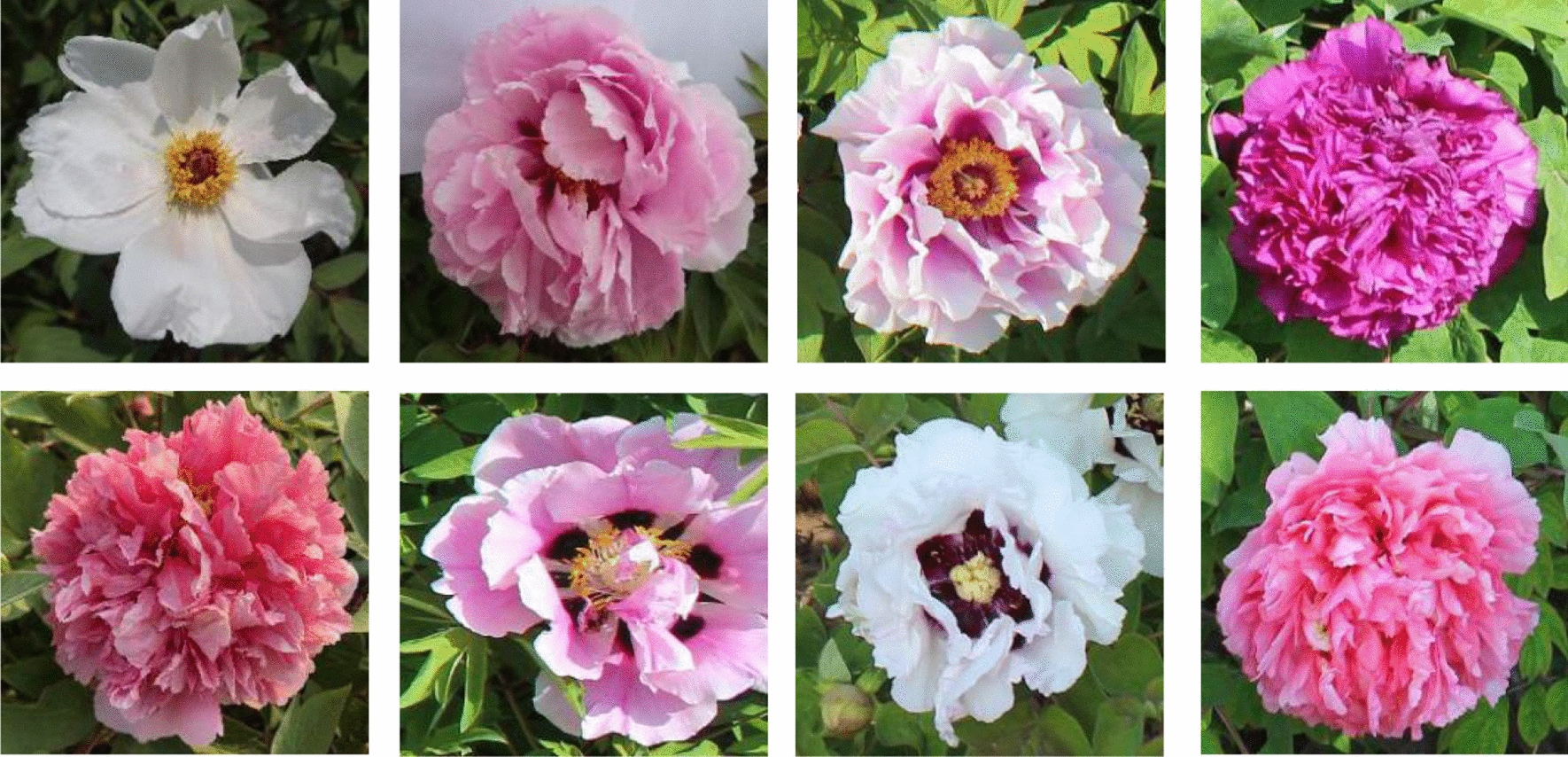
Sample images in peony dataset

Peonies were cultivated in a natural environment, the images were captured by the researchers with a digital camera under natural light in 2019. There are 80 cultivars of peonies, each of which contains 3-40 images, for a total of 1255 images. Due to the nonuniform background and the different shooting angles of these images, manual shooting is more subjective and flexible than shooting by a machine. Besides, there may be multiple peonies in one picture. Since the task of flower cultivars identification requires a complete top-view of images, and each image contains only one cultivar, the original images need to be annotated and cropped. We stored the cropped images by cultivars, checked each image manually, and screened out the images with overexposure, incomplete patterns, and poor definition to meet the requirements of the identification task. By random cropping, we expanded the number of original images by 10 times to a total of 12550. We selected 20 images for each cultivar from the augmented images randomly, removed cultivars with less than 20 images, and finally constructed a balanced dataset containing 11200 images of 56 cultivars. Some of the images were shown in Fig. 4. 80% of the images were used for training and 20% for test.

### Oxford 102 flowers dataset

**Fig. 5:**
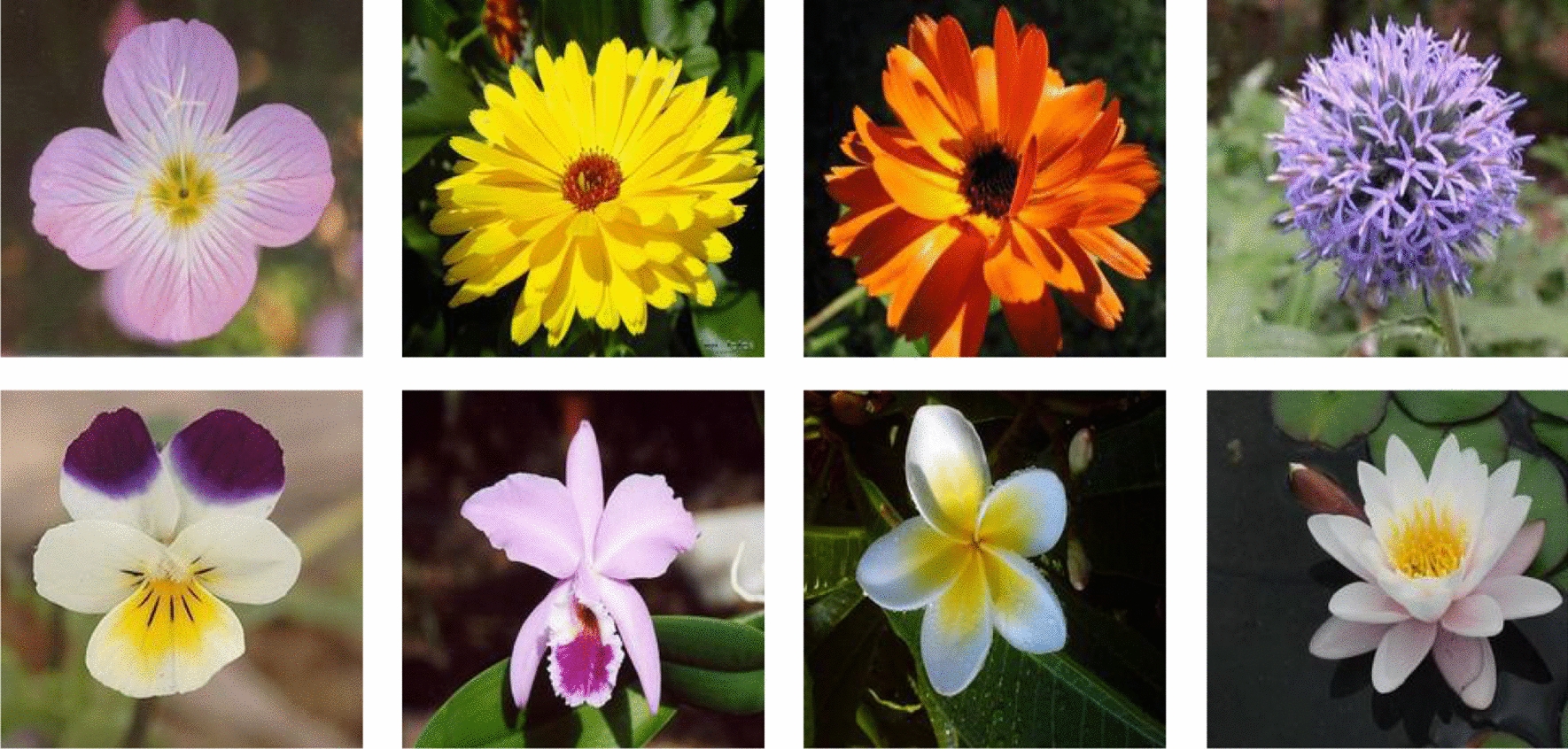
Sample images in Oxford 102 Flowers dataset

Oxford 102 Flowers dataset (Fig. 5) is a flower collection dataset released by the Department of Engineering Science of Oxford University in 2008 [24]. It is mainly used for image classification, consisting of 102 different classes of flowers common to the UK, each class contains 40 to 258 images. Since the flower cultivars identification task requires a complete top view of the flower, we first selected 3384 images of 80 classes of the original Oxford 102 Flowers dataset. By random cropping, we expanded the number of original images by 10 times and then randomly select 20 images for each class from the augmented images, remove classes with less than 20 images, and finally construct a balanced dataset containing 15400 images of 77 classes. Likewise, 80% of the images were used for training and 20% for test. Table 1 shows the details of the three datasets.

**Table 1:** Details of three datasets

Dataset	Original dataset		Augmented dataset	
	No. of classes	No. of images	No. of classes	No. of images
Chrysanthemum dataset	126	2520	126	25200
Peony dataset	80	1255	56	11200
Oxford 102 Flowers dataset	102	8189	77	15400

### Devices

The models were built and trained on the Ubuntu 16.04 system, based on Intel Xeon Gold 5120 CPU and 4 NVIDIA Titan Xp 16GB GPU hardware platform. PyTorch was used for our experiments.

### Metric learning method

**Fig. 6:**
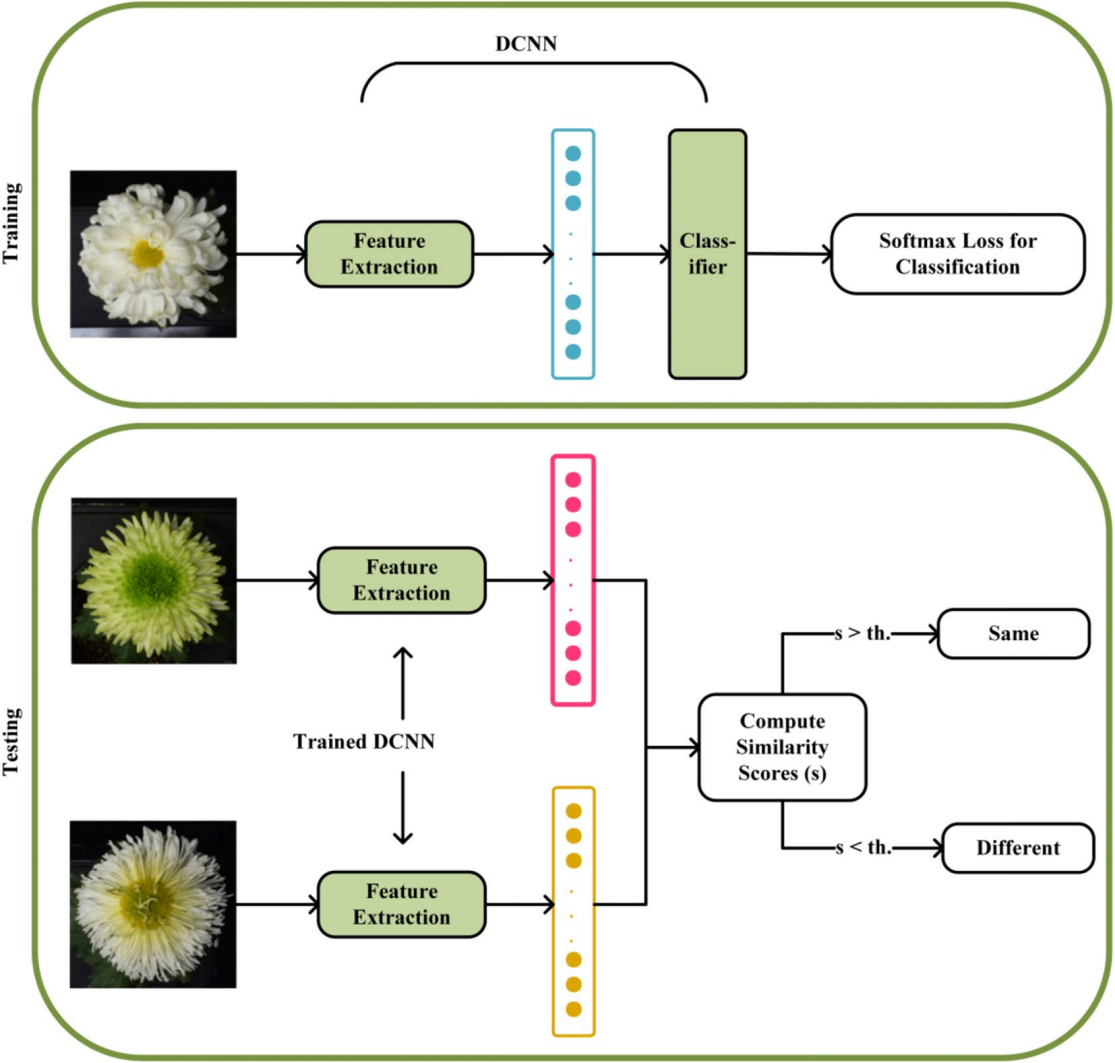
The general pipeline for training a chrysanthemum cultivars identification system using DCNN

Refer to the method of [22], we first summarize the general pipeline for training a chrysanthemum cultivars identification system using DCNN as shown in Fig. 6. The DCNN includes a feature extractor and classifier. During training, the DCNN is trained where the network learns to classify a given chrysanthemum cultivars image patch to its correct identity label, a softmax loss function is used for training the network which is given by Eq. (1)

1 $${L_S} = - \frac{1}{M}\sum\limits_{i = 1}^M {\log \frac{{{e^{W_{{y_i}}^Tf({x_i}) + {b_{{y_i}}}}}}}{{\sum\nolimits_{j = 1}^C {{e^{W_j^Tf({x_i}) + {b_j}}}} }}}$$

where *M* is the training batch size, ${x_i}$ is the* i*th input chrysanthemum cultivars image patch in the batch, feature descriptor $f({x_i})$ is the corresponding output of the feature extractor, ${y_i}$ is the corresponding class label, and *W* and *b* are the weights and bias for the last layer of the network which acts as a classifier. Feature descriptor $f({x_i})$ is also the embedding vector using the *L*_*2*_-norm.

At test time, the embedding vectors $f({x_g})$ and $f({x_p})$ are extracted for the pair of test chrysanthemum cultivars images ${x_g}$ and ${x_p}$ respectively using the trained DCNN, and normalized to unit length. Then, a similarity score *s* is computed as the *L*_2_-distance or using cosine similarity, as given by Eq. (2). Due to normalized, the two results are equivalent. If the similarity score is greater than a set threshold, the chrysanthemum cultivars pairs are decided to be of the same cultivars.

2 $$s = \frac{{f{{({x_g})}^T}f({x_p})}}{{\parallel f({x_g}){\parallel _2}\parallel f({x_p}){\parallel _2}}}$$

To ensure that samples of the same cultivars are as close as possible during training, center loss [21] was proposed to enhance the discrimination of the deep features trained by DCNN, which can simultaneously learn a center for deep features of each class and minimize the distances between the deep features and their corresponding class centers. Center loss can efficiently pull the deep features of the same class to their centers to improve the similarity. In this paper, the total loss function includes center loss and *L*_2_-softmax loss [22], which can increase the distance between different samples while reducing the distance inside the same samples so that the learned features have better generalization and discrimination ability to improve the feature recognition ability of DCNN. The total loss function is defined as

3 $$L = {L_S} + \lambda {L_C}$$

4 $${L_C} = \frac{1}{2}\sum\limits_{i = 1}^m {\parallel {x_i} - {c_{{y_i}}}\parallel _2^2}$$

and *L*_S_ is *L*_2_-softmax loss, *L*_C_ is center loss, $\lambda$ is a scalar to balance the two loss functions, ${c_{{y_i}}} \in {\mathbb{R}^d}$ denotes the ${y_i}$ th class embedding center vector in embedding space.

**Fig. 7:**
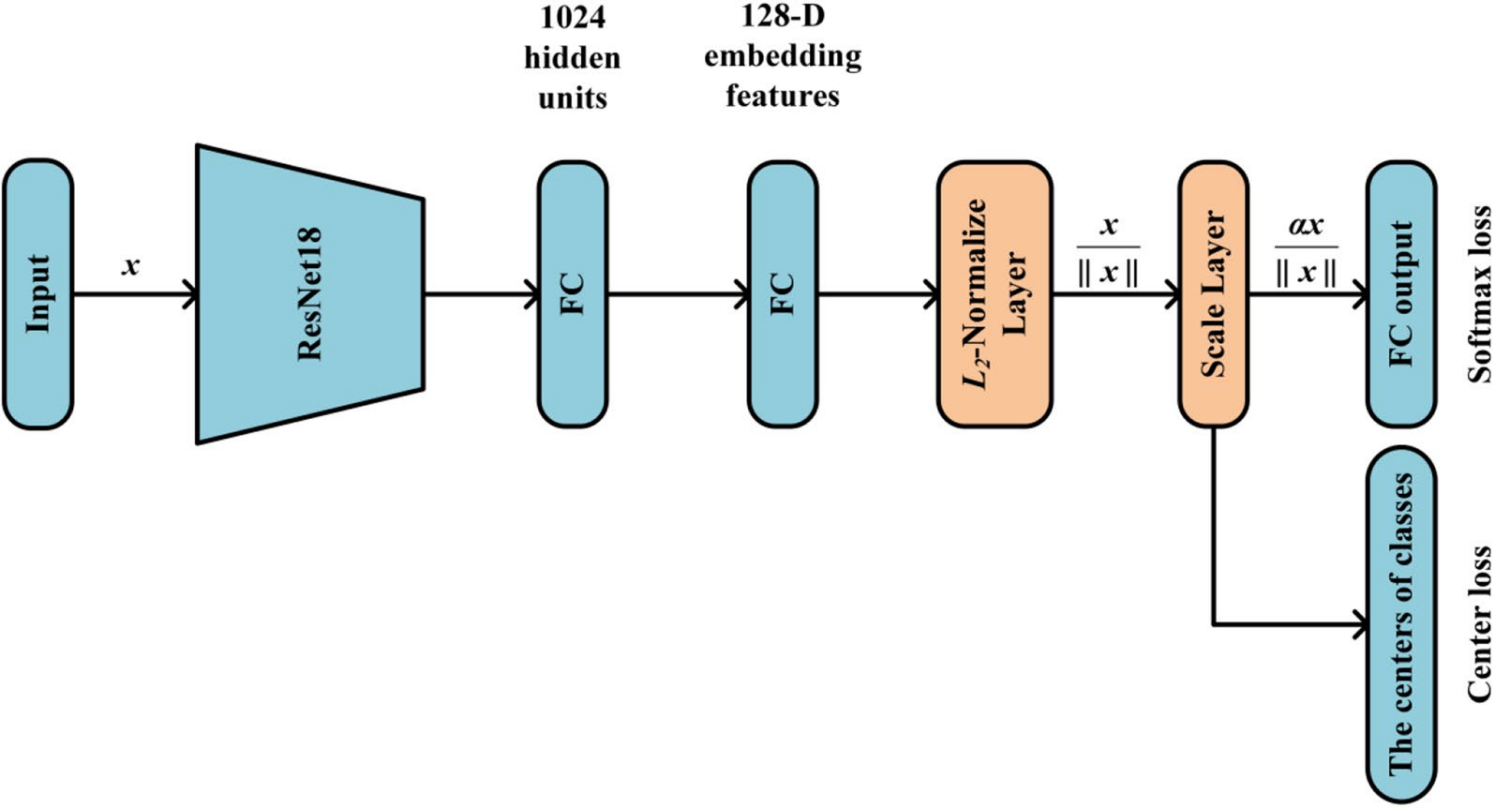
The network architecture. FC represents the fully connected layer

A *L*_2_-softmax loss function [20] is given by Eq. (5)

5 $$\eqalign{ & Minimize - \frac{1}{M}\sum\limits_{i = 1}^M {\log \frac{{{e^{W_{{y_i}}^Tf({x_i}) + {b_{{y_i}}}}}}}{{\sum\nolimits_{j = 1}^C {{e^{W_j^Tf({x_i}) + {b_j}}}} }}} \cr & Subject\,to\parallel f({x_i}){\parallel _2} = \alpha ,\forall i = 1,2,...,M, \cr}$$

where ${x_i}$ denotes the input image, ${y_i}$ denotes the corresponding class label, $f({x_i})$ denotes the feature descriptor which is restricted by a constant value $\alpha$. *W* and* b* are the weights and bias for the last layer of the network, the size of mini-batch and the number of classes is *M* and *C*, respectively. Fig. 7 shows the architecture of the network.

### Implementation details

According to [25], ImageNet-trained CNNs are strongly biased towards recognizing textures. But for ornamental plants, color is a very important factor that must be considered. Thus, we used the script of ResNet18 [26] as a feature extractor to learn 128-D embedding instead of using pre-trained on ImageNet [27] dataset for our experiments. Besides, the script of ResNet50 and DenseNet121 were used for comparative experiments on chrysanthemum dataset.

Our model requires the fixed-size (224×224 pixel) input images, so the original images were first scaled to 256×256 pixels, and then images of 224×224 pixels were randomly cropped from the scaled images to obtain different scales and local feature images.

For chrysanthemum dataset, due to the use of the center loss, we adopt P-K sampling strategy [28] to construct mini-batch in training. The core idea is to form mini-batch by randomly sampling P classes (chrysanthemum cultivars), and then randomly sampling K images of each class (cultivar), thus resulting in a mini-batch of PK images. The DCNN was initialized by He initialization [29]. The test set consists of 26 cultivars that are different from the training set, with a total of 5200 sample pairs, 2600 pairs of positive samples, and 2600 pairs of negative samples. All positive and negative pairs were sampled randomly and performed to binary decision and 10 cross-validation. For peony dataset and Oxford 102 Flowers dataset, in terms of dataset division, we performed a random shuffling of the images in the datasets mentioned above, 80% of images were used for training and the rest for test. Others are the same as chrysanthemum.

The model supervised by *L*_2_-softmax loss and center loss trained 30 epochs in total. Using ResNet18, the training time of chrysanthemum dataset and the Oxford 102 Flowers dataset was around 3 hours and was around 2 hours for peony dataset. Using ResNet50 and DenseNet121, the training time of chrysanthemum dataset was around 3 hours and 4 hours, respectively. The Adam optimizer was used to optimize the network with the initial learning rate of 0.001, and the step-decay strategy was adopted for the learning rate adjustment. We fix the hyperparameter $\lambda$ to 1 and $\alpha$ to 10 in this experiment.

### Evaluation

We used Top-k accuracy, which has been widely used to evaluate the DCNN model in image classification, as the evaluation criteria to evaluate our model. The network gives the most likely k classification results, the classification is considered correct if the k results include the correct class. Therefore, we applied the Top-k accuracy to our model and used the average of all images in the test set of each cultivar as the Top-1 and Top-5 accuracy. In addition, we used the receiver operating characteristic (ROC) curve which is commonly used to analyze results for binary decision problems and the area under the ROC curve (AUC) as the evaluation metrics.

T-Distributed Stochastic Neighbor Embedding (T-SNE) is a technique for dimensionality reduction that is particularly well suited for the visualization of high-dimensional datasets [30]. T-SNE is a nonlinear dimensionality reduction algorithm, which is very suitable for dimensionality reduction of high-dimensional data to two or three dimensions for visualization. For points with greater similarity, the distance of t distribution in the low-dimensional space needs to be smaller; for points with low similarity, the distance of t distribution in the low-dimensional space needs to be farther. This just satisfies our need for features in the same cluster (closer distance) to gather closer, and features between different clusters (farther distance) to be more distant. For the above methods, we extracted high-dimensional features of the training set and test set images respectively and visualized them in a two-dimensional space.

## Results

### Model accuracy performance on chrysanthemum dataset

For joint supervision of center loss and *L*_2_-softmax loss on chrysanthemum dataset, two P-K sampling schemes were designed. For each batch of training, one scheme includes *P*=5, *K*=10, and a total of 50 images; the other includes *P*=18, *K*= 5 images, and a total of 90 images. Table 2 lists the Top-1 accuracy (%) of chrysanthemum test set obtained with the two schemes for different network architecture. ResNet18 with P=18, K=5 achieves the highest performance by the accuracy of 91.88%.

**Table 2:** Top-1 accuracy (%) of chrysanthemum test set

Model	Acc.
ResNet18 (P=5, K=10)	89.50
ResNet18 (P=18, K=5)	91.88
ResNet50 (P=5, K=10)	66.21
ResNet50 (P=18, K=5)	90.54
DenseNet121 (P=5, K=10)	89.33

### The ROC curve on chrysanthemum test set

**Fig. 8:**
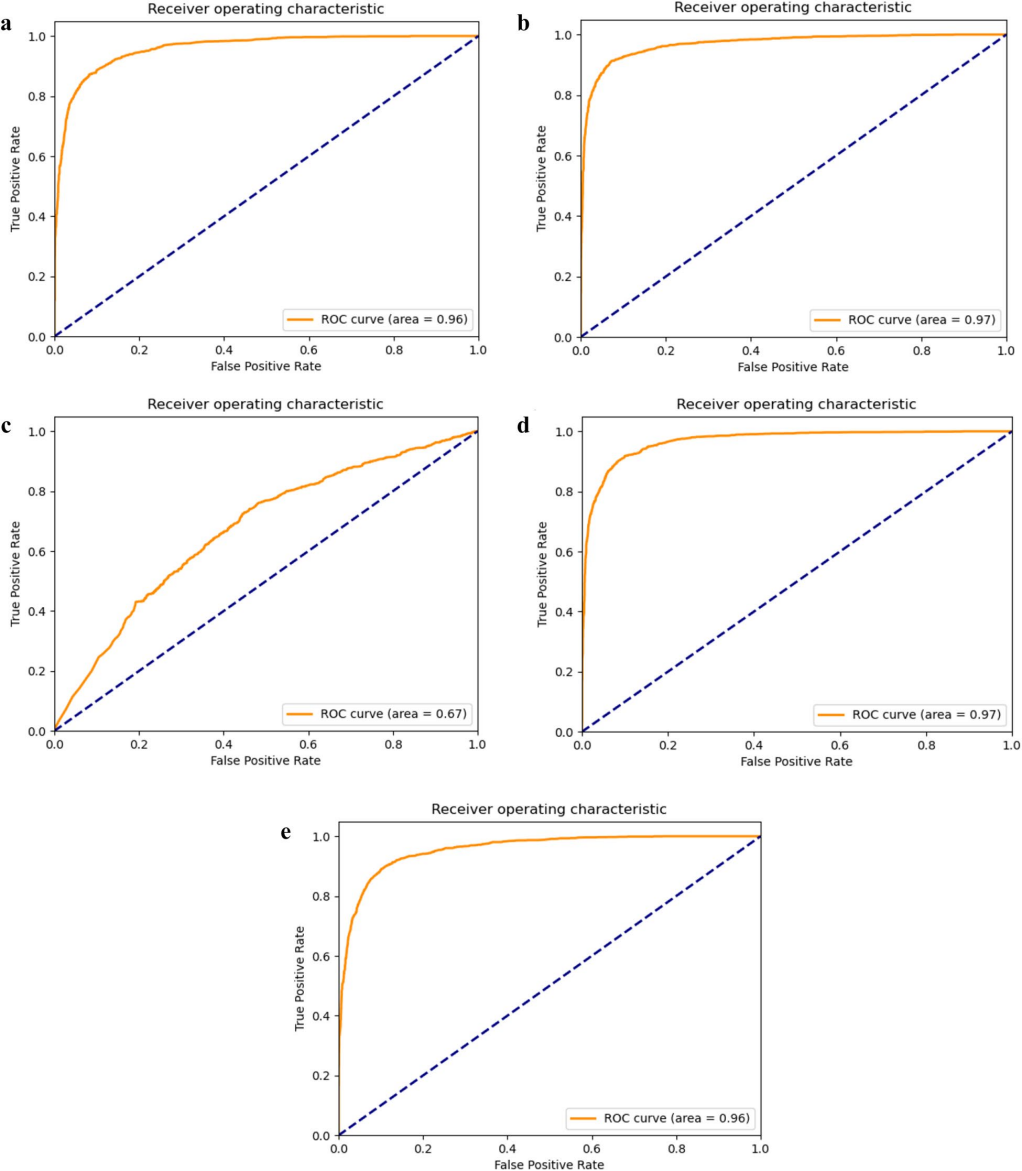
ROC curve of chrysanthemum test set. **a** ResNet18 (P=5, K=10). **b** ResNet18 (P=18, K=5). **c** ResNet50 (P=5, K=10). **d** ResNet50 (P=18, K=5). **e** DenseNet121 (P=5, K=10)

The ROC curve of the five models joint supervision of center loss and *L*_2_-softmax loss after 10 cross-validations on chrysanthemum test dataset are shown in Fig. 8.

### T-SNE visualization on chrysanthemum dataset

**Fig. 9:**
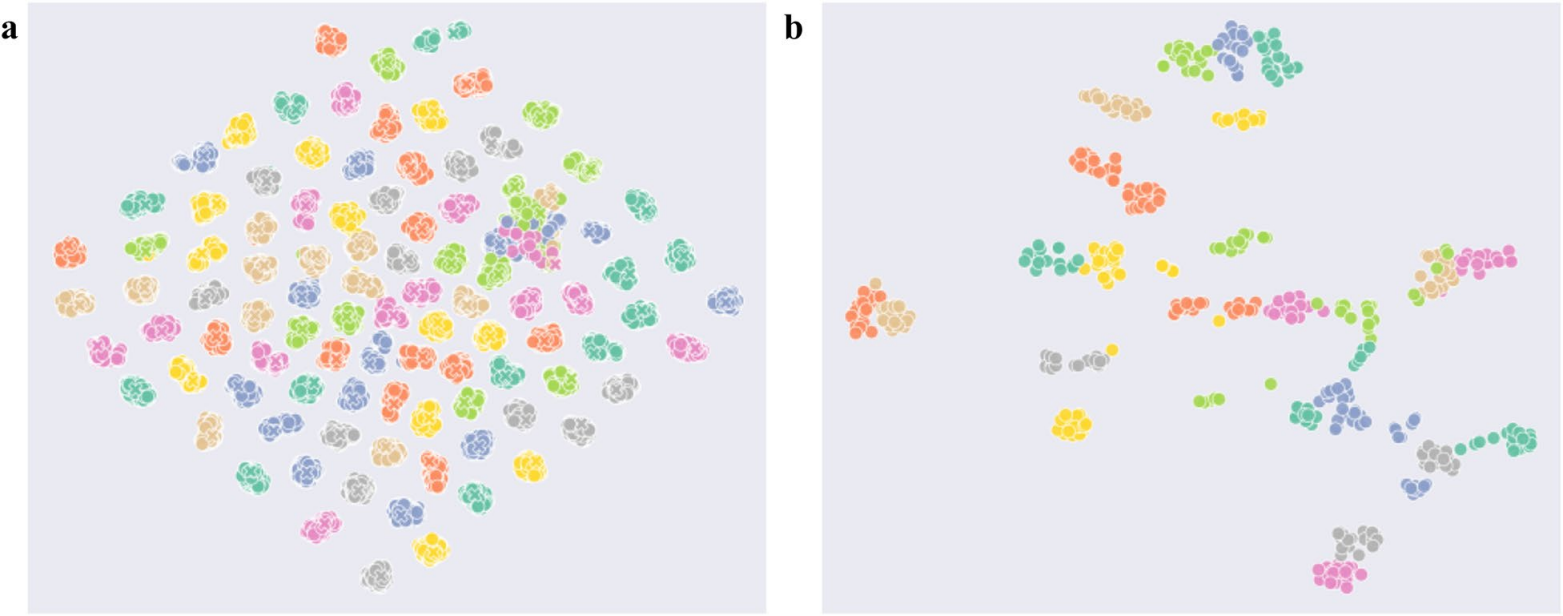
Dimension-reduced scatter plot of ResNet18 (P=18, K=5) on chrysanthemum dataset. **a** The feature distribution of the training set, × represents the center of each cultivar. **b** The feature distribution of the test set

**Fig. 10:**
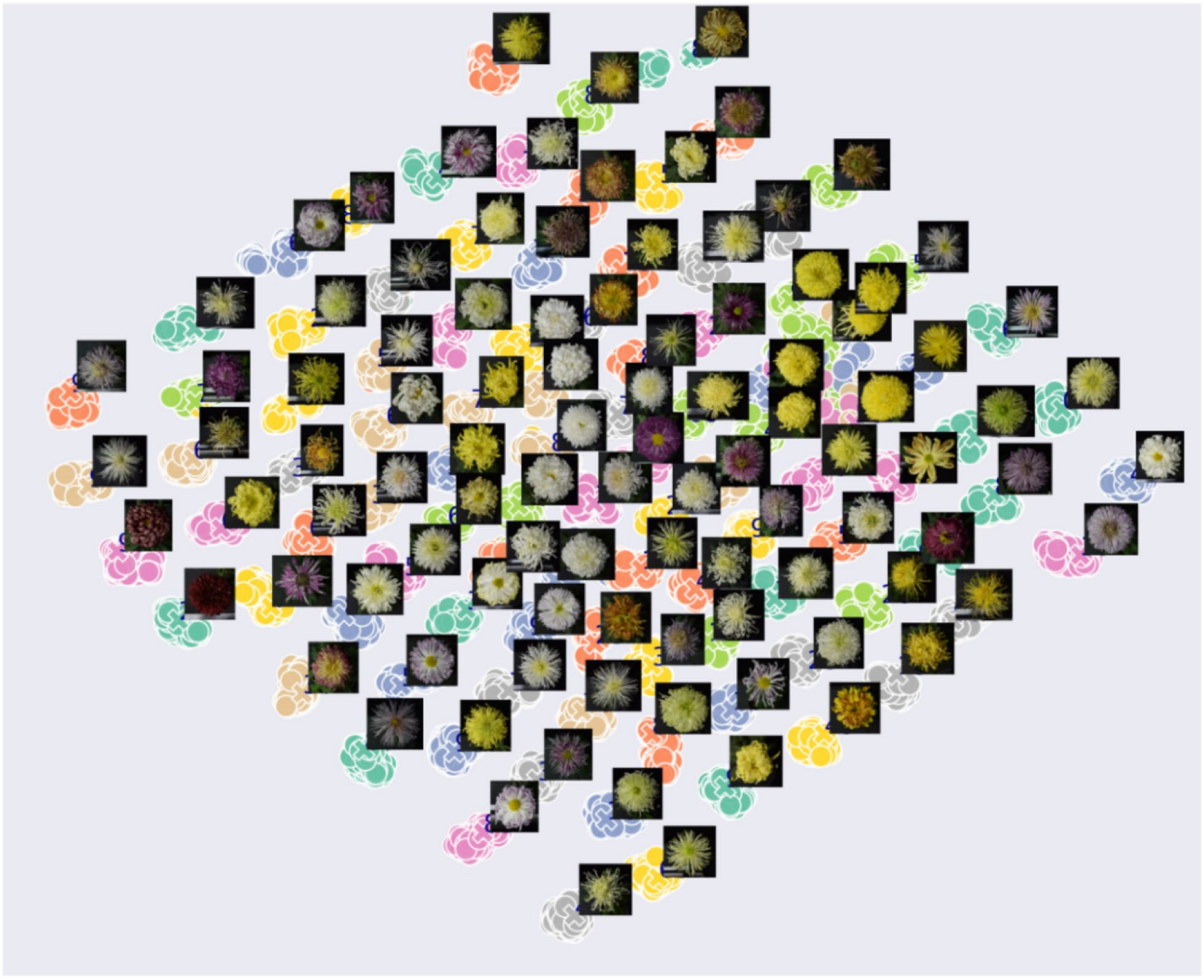
The feature distribution of some example images of each cultivar in the training set. × represents the center of each cultivar

**Fig. 11:**
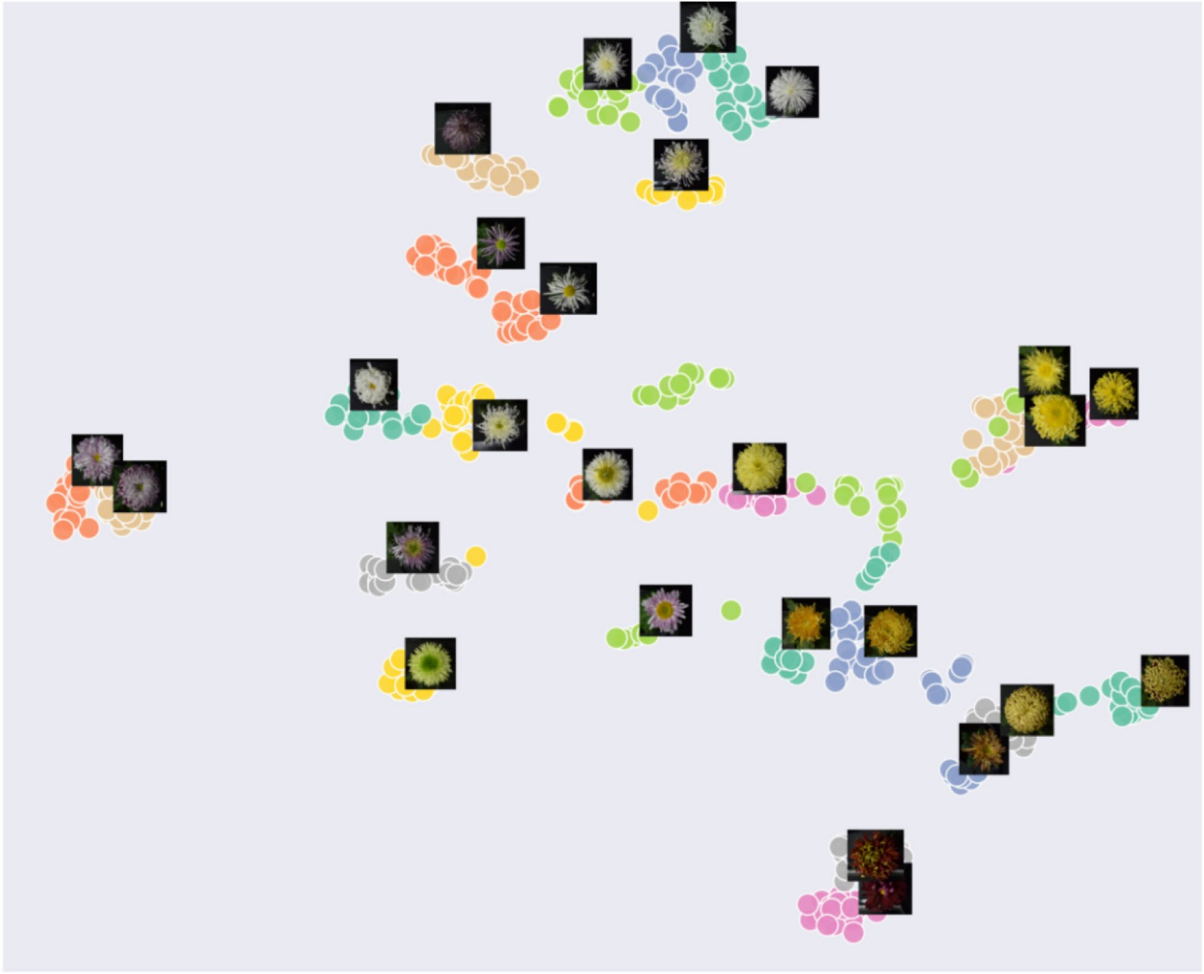
The feature distribution of some example images of each cultivar in the test set

To directly observe the distribution of features extracted by the model about different cultivar images, we applied T-SNE nonlinear dimensionality reduction algorithm. The dimension-reduced scatter plot (Fig. 9) shows the distribution of features extracted from images on chrysanthemum dataset. Fig. 9a depicts the 2-dimension features reduced from 128-dimension in the training set. We can see from this figure that most of the images are mapped on their own fixed areas, images of chrysanthemums from the same cultivar are grouped together, different cultivars are separated, but there is overlap in a small area. By observing the feature distribution map along with example images of each cultivar in Fig. 10, we find that the chrysanthemum in the overlapping area has the same color and similar morphology. This phenomenon also appears in the test set as shown in Figs. 9b and 11.

### Results on peony dataset and Oxford 102 flowers dataset

Top-1 and Top-5 accuracy rates are used to evaluate the model trained with only *L*_2_*-*softmax loss (Model S) and joint supervision of center loss and *L*_2_*-*softmax loss (Model C) on peony dataset and Oxford 102 Flowers dataset. Both models include *P*=18, *K*= 5 images, and a total of 90 images. Table 3 shows the accuracies of peony dataset. With and without center loss, the model both achieved high recognition accuracy rates. Top-1 accuracy rates of the model are 93.97% and 97.99%, respectively, and Top-5 accuracy rates of the model are 99.82% and 99.73%, respectively. In Table 4, Top-1 accuracy rates of Oxford 102 Flowers dataset with and without center loss are 86.13% and 95.36%, respectively, and Top-5 accuracy rates are 97.34% and 99.42%, respectively, which are slightly lower compared to peony dataset.

**Table 3:** Top-K rates (%) of peony dataset

Model	Top-1 acc.	Top-5 acc.
Model S	97.99	99.73
Model C	93.97	99.82

**Table 4:** Top-K rates (%) of Oxford 102 Flowers dataset

Model	Top-1 acc.	Top-5 acc.
Model S	95.36	99.42
Model C	86.13	97.34

**Fig. 12:**
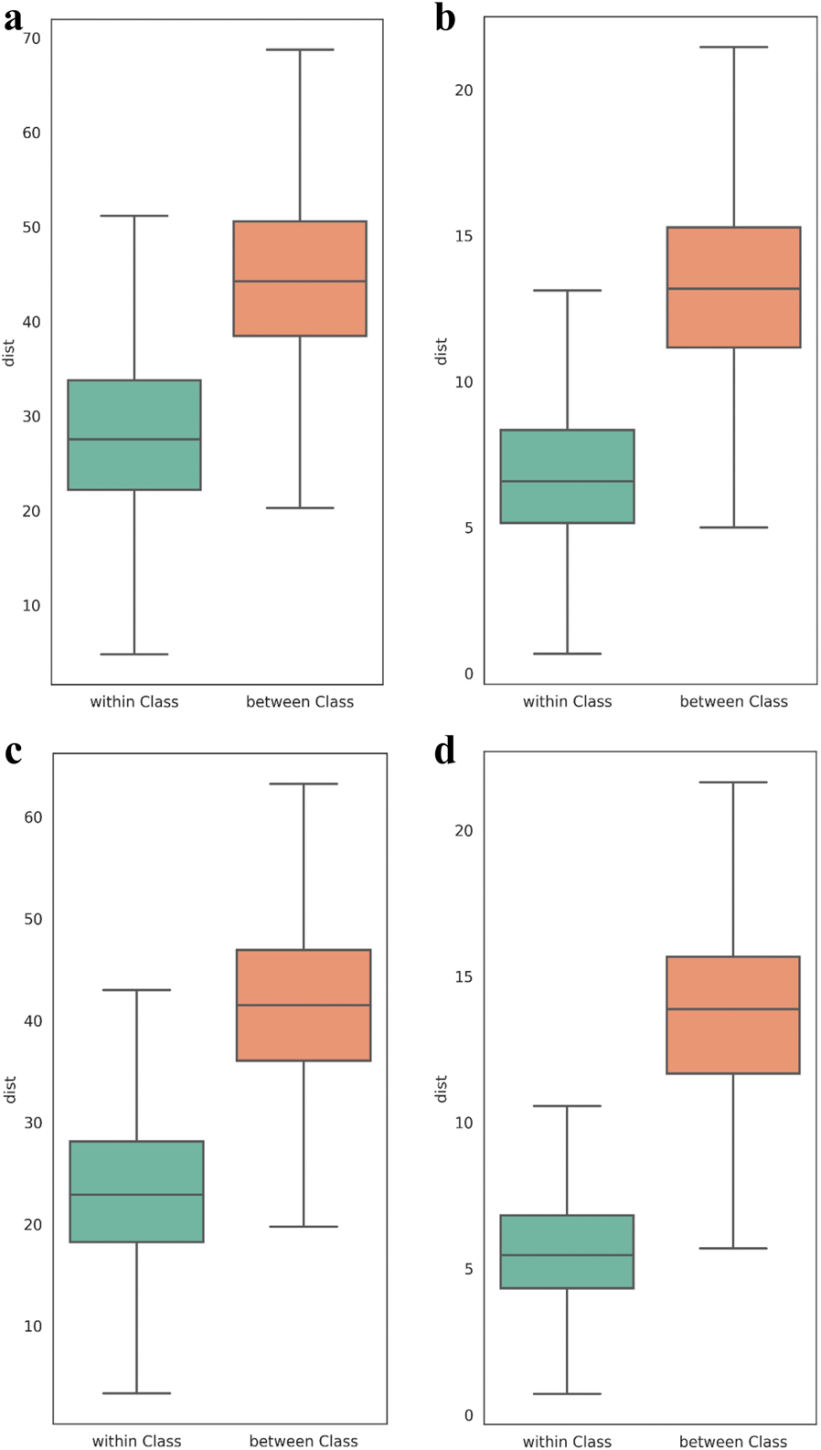
The distance boxplot. Note: **a** The distance boxplot of the Oxford 102 Flowers dataset of Model S. **b** The distance boxplot of the Oxford 102 Flowers dataset of Model C. **c** The distance boxplot of peony dataset of Model S. **d** The distance boxplot of peony dataset of Model C

To analyze the distribution of features, we drew the boxplots to reflect the distance within and between classes. The distance boxplots are shown in Fig. 12a, b are the boxplots of the Oxford 102 Flowers dataset, Fig. 12c, d are of peony dataset. Among them, Fig. 12a, c only use *L*_2_-softmax loss, Fig. 12b, d joint supervision of center loss and *L*_2_-softmax loss. The boxplot on the left of each figure represents the distance within the class, and the boxplot on the right represents the distance between classes. The upper and lower lines in the boxplot represent the maximum and minimum values of the data, respectively. The upper and lower boundaries of the rectangular box represent the upper and lower quartiles of the data, respectively, and the middle line represents the median of the data. Compared with the model that only uses *L*_*2*_-softmax loss, the model that adds center loss can reduce intra-class distance significantly.

## Discussion

Deep learning and other related technologies have been applied to the research of flower classification tasks and made a series of important progress [10–13]. Although these methods achieved high recognition accuracy, the image features obtained through the classification network do not reflect the botanical features of flowers, which cannot provide further help for botany research. Compared to only identifying plant species from flower images [10], flower cultivars identification based on metric learning can not only classify flower cultivars accurately, but more importantly make the model has the ability to metric and make the classification results interpretable. In this study, we proposed a method based on metric learning by adding center loss to the classification network for flower cultivars identification. Three different and representative datasets are chosen to evaluate our proposed method and validated the effectiveness.

The model trained in combination with center loss and *L*_2_-softmax loss on chrysanthemum dataset, for adopting P-K sampling strategy to construct mini-batch in training, the accuracy of the model using P=18, K=5 scheme is higher than that of using P=5, K=10. This shows that the more classes are included in each batch, the training will be faster and the test accuracy will improve. Top-1 accuracies of different feature extraction networks show that the accuracy rates do not blindly increase as the number of network layers deepens. Due to the small amount of data in this research, there may be overfitting problems on networks with deeper layers. Therefore, ResNet18 has the highest Top-1 accuracy under the same P and K values among the three different network architectures. For peony dataset and Oxford 102 Flowers dataset, we found that the model joint supervision of center loss and *L*_2_-softmax loss has a slightly lower accuracy compared to only using *L*_2_-softmax loss, but the feature of the same class gathers more closely. Unlike the image angles of peony dataset, which are mostly top-view and oblique-view, Oxford 102 Flowers dataset has a variety of image angles, so the selection of its feature center is slightly difficult, resulting the clustering within the class is not as obvious as peony dataset. Different from face recognition, the backgrounds of the images in peony dataset and Oxford 102 Flowers dataset are not uniform, so the optimization effect will be weakened.

DCNN model is difficult to interpret, visualizing the outputs helps us to understand its training results [31]. The visualization results show that after training, image patches from the same cultivar are gathered on the feature center representing the cultivar, achieving the purpose of establishing cultivar features through local information. In addition, we found that the chrysanthemums in the overlapping area have the same color and similar morphology, while the color and morphology information was not provided during the training, which shows that the network has indeed learned the potential botany information from the image. Especially in the test set, although the chrysanthemum cultivars in the test set did not appear in the training set, the model can still distinguish the unseen cultivars when the test set is applied to the model, indicating that the features learned by the model have good discrimination ability, as compared to results reported in [14]. The model has achieved high generalization, which proves the effectiveness of metric learning. Center loss only focuses on reducing intra-class distance and does not deal with the inter-class distance, which leads to overlaps between different classes. Since each mini-batch updates the center once during the training process which will make the center unstable, it needs to be combined with the *L*_*2*_-softmax loss to maintain stability. Center loss is mostly used for face recognition in previous research [21], so how to solve the overlap problem between samples of different cultivars to make it more suitable for flower cultivars identification and make the model more universal are the direction of our future research.

## Conclusions

In this paper, we developed a metric learning method based on DCNN for flower cultivars identification tasks. The results have shown the effectiveness of the proposed method with high recognition rates and the feature extracted from the recognition network is interpretable. The same class gathers more closely and has a strong aggregation, and the distance between different classes increases, showing stronger separation. This study can provide new ideas for the application of a small amount of data in the field of identification, and has important reference significance for the flower cultivars identification research.

## Data Availability

The datasets that were used and analysed during the current study are available from the corresponding author upon reasonable request.

## Abbreviations

AI: Artificial intelligence
CNN: Convolutional neural networks
DCNN: Deep convolutional neural networks
FSL: Few-shot learning
SGD: Stochastic gradient descent
ROC: receiver operating characteristic
AUC: area under ROC curve
T-SNE: T-distributed stochastic neighbor embedding
